# “Do they think I’m good enough?”: General practitioners’ experiences when treating doctor-patients

**DOI:** 10.1186/s12875-024-02592-1

**Published:** 2024-09-16

**Authors:** Claire J. Hutton, Margaret Kay, Penny Round, Chris Barton

**Affiliations:** 1 Department of General Practice, School of Public Health and Preventive Medicine, Monash University, 553 St Kilda Road, Melbourne, 3000 Australia; 2 General Practice Clinical Unit, Faculty of Medicine, University of Queensland, Brisbane, Australia; 3 School of Curriculum Teaching and Inclusive Education, Faculty of Education, Monash University, Melbourne, Australia

**Keywords:** General practitioners, Physicians, Family, Physician’s role, Physician-patient relations, Physicians, Practice patterns, Grounded theory

## Abstract

**Background:**

When doctors seek medical care, there is evidence that the treating doctor can struggle to provide optimal treatment. Guidelines state that doctor-patients should be treated like any other patient, but this is challenging for the treating doctor. This study set out to explore both the positive experiences general practitioners (GPs) have when caring for doctor-patients, and the challenges they confront. It sought to identify whether GPs believe they treat doctor-patients differently to other patients and if so, in what ways, for what reasons, and how this impacts their provision of care. The study also aimed to develop a model that makes sense of GPs’ experiences when caring for a patient who is also a medical doctor.

**Method:**

Qualitative in-depth interviews with 26 GPs were carried out, with analysis of de-identified transcripts using pragmatic grounded theory. Evolving understandings were used to develop a model to make sense of GPs’ experiences caring for their doctor-patients.

**Results:**

The core aspects of GPs’ experiences of treating fellow doctors centred around concepts of respect and collegiality. These play a central role in mediating how a treating doctor experiences a consultation with a doctor-patient, influencing the quality of care provided. GPs shared that the use of medical language (and assumptions about the doctor-patient’s knowledge/behaviours), testing, the exploration of sensitive issues, and the degree of shared decision-making were areas where their treatment might vary when treating a doctor-patient. Treating doctors often experience anxiety about errors and the likely scrutiny from the medical, and wider community. The decision to treat the doctor-patient differently was driven by a desire to maintain a sense of collegiality, to not offend, to meet their doctor-patient’s expectations, and to appear competent.

**Conclusion:**

The professional socialisation of doctors, with its emphasis on collegiality and respect, plays a significant role in the dynamics of the therapeutic relationship when a doctor treats a doctor-patient. Current guidelines make little reference to these dynamics with the over-simplified ‘keep it normal’ recommendations. Treating doctors need evidence-informed training to navigate these challenges and ensure they can effectively deliver quality care to their doctor-patients.

**Supplementary Information:**

The online version contains supplementary material available at 10.1186/s12875-024-02592-1.

## How this fits in:

- Previous studies on treating doctor-patients have identified maintaining boundaries and avoiding assumptions about the doctor-patient’s knowledge/behaviours as key challenges.
- Guidelines state that doctor-patients should be treated like other patients, but fail to acknowledge the dynamics that make adherence to ‘normal’ practice challenging for the treating doctor.
- This study highlights the importance treating doctors place on maintaining collegiality and demonstrating respect when providing medical care to doctor-patients.
- It details the challenges in adhering to these values while maintaining boundaries, and their impact on the quality of care delivered.

## Introduction

Doctors’ wellness is strongly associated with the delivery of high-quality patient care [1]. Yet, doctors often delay seeking medical care for themselves. Despite the strong recommendations of medical regulators that doctors have their own (independent) general practitioner (GP), many do not [2]*.* A literature review [2] investigating barriers to health access for doctors identified provider barriers as a significantly under-researched area.

Morishita et al. [3] reviewed the body of literature exploring the experience of the doctor-patient. These understandings provide some insights on the delivery of care. Personal narratives [4–6] together with qualitative research [7] have demonstrated how poor care contributes to the barriers that doctors experience when seeking medical care.

The provider of care, the treating doctor, is an important component that determines the quality of medical care that a doctor receives. The experience of the treating doctor when the patient is a doctor remains poorly understood. Kay [8] reported that many doctors are ambivalent about accepting another doctor as their patient, though empirical data are lacking.

Guidelines state that doctor-patients should be treated like any other patient [9, 10], however this appears to be difficult to put into practice. A scoping review [11] revealed a predominance of expert opinion articles, and few empirical studies to guide doctors treating another doctor. These studies describe anxiety about missing something or being criticised (leading to over-testing) and concern about upsetting/offending the doctor-patient. These responses could result in the avoidance of sensitive discussions about mental health, or alcohol and other drug use. Boundary issues such as over-identifying with the doctor-patient, treating them more like a colleague rather than as a patient, or making assumptions about the doctor-patient’s level of knowledge and not providing adequate information were other themes that were identified.

Overall, the studies included in this review suggest that treating doctors may struggle to deliver optimal care to their doctor-patient, but offered few answers as to *why* both their experience and their approach might differ significantly when their patient is a doctor.

Many doctors, and especially general practitioners (GPs), will find themselves treating fellow doctors. If some doctors do find treating doctor-patients anxiety-provoking and challenging, then a greater understanding of the complex relationship between treating doctor and doctor-patient is needed. This will enable doctors to receive the same high-quality care as other patients.

To address this gap in knowledge, this study asked GPs about their experiences when treating doctor patients. By exploring these complex issues, this study also aimed to develop a model, grounded in the experience of doctors, to guide practice and the provision of quality care to doctor-patients.

## Methods

### Study design and setting

A qualitative study was conducted involving in-depth interviews with GPs in Australia. We chose to focus on GPs, as this is the entry point to the medical system in Australia, where GPs have the dual role of providing primary health care, and as the gatekeeper for entry to specialist care. A pragmatic grounded theory [12] study design was adopted, as the area of research is exploratory, and there is no current theory to explain or understand these processes. The philosophical underpinnings of pragmatism, that the useful or practical is privileged over the theoretical [13], were appropriate as the goal was to develop a theory from which specific recommendations for practice could be drawn.

### Research team

The primary author (CH), a psychologist with 30 years’ experience, carried out all interviews, listened to the audio recordings, verified the transcripts, and collaborated with the co-authors (CB, MK, and PR) to compare codes and develop categories as part of her doctoral research. CB is an experienced primary care academic and qualitative researcher who studies patient and GP experiences of care. MK is a general practitioner and doctors’ health researcher with extensive experience supporting the wellbeing and care of doctors. PR is an experienced academic in Education, with experience in qualitative research methodology.

### Participants and Sampling

Initially, purposive sampling was used, selecting participants who had experience treating other doctors, in order to select an information rich sample who could answer the research question [14]. As the study progressed, theoretical sampling [15, 16] (following leads in the data by including new participants who can provide relevant information) was used, to better understand the emerging theory. For example, the opinions of additional male doctors were sought as they generally have higher levels of clinical self-confidence than female doctors [17]. Similarly, additional sampling of GPs in their mid-career and beyond occurred, as one of the few empirical studies in this area found more experienced primary care physicians were less likely to report anxiety when caring for doctor-patients [18]. The sample size was guided by the concept of information power [19], which emphasises the quality of the interviews, as well as the aims of the study, specificity of the sample, the use of theory and the strategy for analysis in making decisions about sample size. Theoretical sampling [16] guided the decision to end recruitment, when the exploration of categories and concepts was exhausted.

Participants were recruited via a range of approaches including posts to professional organisation websites, noticeboards and newsletters (including AMA Victoria, Australasian Association for Academic Primary Care, the Royal Australian College of General Practitioners, University Departments of General Practice), and an Australian Facebook group for doctors (GPs Down Under).

Participants were given an Explanatory statement (which specified that the study formed part of CH’s doctoral research) and provided written informed consent prior to interviews. These took place at a location of the participant’s choosing (their home, their workplace, interviewer’s workplace), or using an online platform. CH’s long experience as a psychologist influenced her preference to carry out interviews face-to-face where possible, believing this facilitates the rapport and trust crucial to a productive interviewer-participant relationship. The online platform was used if the participants’ location was outside Melbourne, or their preferred option was online. Participants were offered a $150 gift voucher in recognition of their time.

### Data collection

One-on-one in-depth interviews used a semi-structured interview guide (Supplemental Material 1), based on Minichello et al.’s [20] approach, and constructed around a series of broad thematic areas to be explored. Topics were informed by the scoping review of the literature [11] and included:

- what GPs find challenging, and rewarding, about treating a doctor-patient
- whether GPs believe they treat doctor-patients differently to other patients and if so, in what ways and for what reasons, and what this means for provision of care.
- advice they would give to a new GP treating a doctor-patient for the first time

Participants were encouraged to add what was important to them about their experience. The interview guide was piloted with the first five GPs (who were known to CH, as members of the same university department), and these interviews were retained for analysis. After completing the pilot interviews, the authors met to review the transcripts and reflexive notes made by CH and discuss how the interviews could be expanded or re-focussed.

Interviews were organised in three rounds, between November 2022 and February 2023. This “zig-zag” approach involved going out to the field to gather information, back to the office to analyse, then back to the field to gather more information, informed by the earlier rounds of interviews and the emerging theory [14].

Interviews were audio-recorded and transcribed verbatim. The first five were transcribed by CH to aid immersion in the data and the remainder by a professional transcription service. Transcripts were imported into NVivo 12 (QSR International Pty Ltd, 2018) for data storage and management and to aid coding. Audio files were transcribed as soon as possible after the interview to allow a process of constant comparison. As the theory emerged from the data, the interview guide was adapted to re-work questions to enhance their clarity, and to explore emergent concepts not previously considered [21]. Increased theoretical sensitivity [22] (the ability to know when you identify data important to the emerging theory) also resulted in concepts like collegiality and respect being explored in more depth by the interviewer, when they arose.

### Data analysis

Data analysis followed the approach of Corbin and Strauss [16]. First, open/initial coding (word-by-word, line by line analysis questioning the data to identify concepts and categories) was undertaken which was followed by intermediate (or axial) coding to transform the basic data into more abstract concepts, and grouping codes into themes. The final stage was theoretical coding, a more refined level of coding that identified relationships between categories developed in the earlier levels of coding, helping to tell an analytic and coherent story [23] (Coding tree excerpts in Supplemental material 2).

Grounded theory uses abductive reasoning which begins with an examination of the data, and the formation of a number of hypotheses that are then proved or disproved during the process of analysis [15]. Constant comparative analysis [12] was undertaken, with new data being compared with data obtained during previous interviews in a linear fashion throughout the data collection phase. New codes were added as interviews were completed, and past interviews were re-coded where necessary*.* Memos were written throughout the analysis to assist with development of axial and selective coding.

Field notes and memos were also used to document the emotional responses, personal biases and experiences the interviewer brought to the interviews and analysis. These biases and experiences were influenced by her position as a psychologist (which contributed to the lens with which she viewed the data). Her long-term involvement in doctors’ health, training and supervising doctors who volunteer on an anonymous peer support service enhanced her ability to understand the perspectives of the participants. This role could contribute to being seen as an ‘insider’, with its double-edged sword of familiarity with the issues, and subjectivity. However, as the interviewer was not a doctor, it is likely to have ensured a more neutral environment for data collection. Not being a peer may have made it less likely that the interviewees would respond with what they perceived to be the ‘correct response’, and may have avoided shared (unspoken) assumptions [24].

Member checking was employed whereby participants were offered the opportunity to review transcripts for accuracy and to clarify or expand upon any points of discussion. Six participants provided responses, three of whom requested that small sections not be used as direct quotations in the final paper, due to concerns the detail might identify them or their doctor-patients.

Once a preliminary theoretical model had been formulated, this was sent to all participants, with a request for feedback about whether the model fits with their experience of treating other doctors. Five participants responded, and their feedback and suggestions further informed the final model.

## Results

Twenty-six GPs participated (16 female, 10 male), with interviews ranging in length from 22 to 60 min (see Table 1).

**Table 1:** Key Characteristics of Participants and Interviews

		*N* = 26 (mean length = 42 min)
Gender	Female	*N* = 16 (ML = 41 min)
	Male	*N* = 10 (ML = 43 min)
Years of experience as GP	1–9	*N* = 12
	10–19	*N* = 5
	20–29	*N* = 2
	30–39	*N* = 4
	40 +	*N* = 3
Location of GP	Victoria (Aus)	*N* = 18
	Other states in Aus / NZ	*N* = 8
Interview method	Face-to-face	*N* = 8 (ML = 46 min)
	Zoom	*N* = 16 (ML = 40.3 min)

The core finding was that GPs believe that they should treat their doctor-patients the same as everyone else, yet the participants went on to describe many ways in which their care was necessarily different. Respect and Collegiality were strong consistent themes emerging from the data. These sit at the centre of the GP’s experience when providing care to fellow doctors.

### How the need to demonstrate respect impacts the consultation

Respect can be understood in different ways by the treating doctor (see Table 2). Some participants saw “respecting my colleagues” as acknowledging their doctor-patient’s prior (self) diagnosis.

*Well I think you have to recognise that your doctor-patient has already done some thinking and (laugh) some diagnosing on themselves before they’ve even come in, so you have to treat that with a little respect, that they probably are a good, and most likely quite accurate historian, or symptom-recognising and possibly even self-treatment issues* (ID01)

**Table 2:** Respect and the Doctor-patient

Impact on the consultation	Exemplar quotes from participants
Respecting the doctor-patient’s views (impact on degree of shared decision-making)	*Because I want to show respect, I maybe can be overly-influenced by my doctor-patient, both about diagnosis, and what the treatment plan should be* (ID08) *As we discuss things […], I might say “Have you seen any new evidence around that?” Or “What’s your professional, what’s your opinion on that?” So really using a lot of shared decision making is very important I think with doctors because they have their own belief system* (ID10) *Look I’d like to say that it doesn’t* [influence how I treat them], *[….] but I’d probably have more of a conversation with them, like you know how thoroughly would you like me to examine you, how thoroughly would you like me to do x, y, z, and I sort of leave it up to them to sort of guide it a bit more* (ID16) *I actually think there’s a level of respect that’s required from like an independent decision-making perspective, so I think offering them to choose like the treatment of the diagnosis, is like a level of disrespect* (ID01)
A shared language	*I think there’s a balance of trying to be respectful and not to talk down on your, any colleagues of ours, that we all work in the same field. But also I think I might have overcompensated and I’m not doing my job properly because I haven’t fully conveyed and haven’t fully spoken my mind so to speak, just because I didn’t want to offend anybody* (ID22) *And in the communication process itself, I’ll be using more medical terms, then for management, it’ll be like, maybe not as much explanation, because, you would sort of think that they know what they’re talking about, like say, the course of duration of taking an antibiotic…* (ID12) *I think that would be disrespectful to explain things, to kind of dumb it down I guess* (ID01) *So I mean you speak in the same level, but you have to be really cautious that you know the same things. So I still speak to a doctor like they don’t know anything, because you can’t really ascertain their level of knowledge into something unless you – you’re quite explicit. And I might say something like “blah, blah, as you may know”. And it might make them feel a bit talked down to. But I still say “as you may know”, because you can’t gauge their level. You can’t – shouldn’t – shouldn’t make any assumption about that…[….] And I never want to put a colleague in a position where they have to ask me what I’m saying– because they’ll never do that!* (ID23)
The impact on history taking (in relation to sensitive areas like mental health and substance use)	*You feel that a health professional would come to you quite clearly if they had concerns of substance misuse or mental health, which is not the best way to clinically practice, but I would say that I would raise the concern of those aspects a lot less. My assumption has been that- they’re a doctor and they’re sort of capable. And they’re working, they’re sort of successful, and so on…And so I- very bad (laugh) on my part. I’ve just realised yes, yes. So I yeah may well have missed- may well have missed times* (ID18) *I suspect there is tempering of what doctors tell their doctors because of that fear [of being reported] and I suspect [….] maybe there would be the temptation to not pry as much for fear of Pandora’s box being opened* (ID03) *Yeah I mean I sort of see the role of a GP as our job to ask those hard questions, but I could imagine alcohol and drug use would be something that you might be (pause) a bit more backward about, but you would assume that they were just the same as you without really knowing that because you overly identify with them. Like you understand quite a lot about their work environment and assume they might have similar social habits* (ID17) *I tend to be very upfront – I would directly ask them that question. And I will explain why too. I might say “so you look a little unwell, some of our colleagues do drink a little bit excessively, do you find yourself in that situation too? Cos I would be concerned if that happened to you, and I would want to help you with that”. I would stress that it’s for their own good, rather than anything else* (ID05)

The degree to which the treating doctor used shared medical language was, for some participants, another way that they demonstrated respect for their doctor-patient. Many expressed concern that ‘dumbing down’ their questions and explanations could be offensive, while a few were willing to take that risk as they believed it was more important to ensure their doctor-patient understood.

Treating a colleague could also result in less exploration of sensitive issues such mental health, or alcohol and other drug use. Reasons for this included concern about offending the patient and potentially jeopardising the collegial relationship, apprehension that the doctor-patient’s responses to such questions could require a medical board notification, and the assumption that a fellow doctor wouldn’t struggle with such issues.

### Apprehension when treating other doctors

Many participants spoke about trepidation when treating another doctor. They worried about whether they were being judged, whether they were good enough, or what might happen if they missed something or made a mistake. They wanted to present as knowledgeable and competent (see Table 3).

*My experience is when I treat a doctor that there is a high expectation by the doctor-patient, and will I be able to achieve that? Will the doctor-patient be satisfied?* (ID15)

**Table 3:** Apprehension when treating other doctors

*Source of anxiety / impact*	*Exemplar quotations*
*Feeling self-conscious / wanting to be seen as competent*	***With doctor-patients generally*** *You become very self-conscious in a doctor-doctor consultation about the way you consult or whether you’re doing a good job, you’re more self-conscious than you would be in a normal consultation, in my experience, so you just think ‘ohh I wonder what they thought about how I explained this’ or ‘I wonder whether they think I’m a good doctor [….] I think there’s a sense that you’re being assessed on your performance in a way that’s different to a normal consultation (ID04)* *As someone who’s gone through the training recently, I don’t think it is very talked about how we approach other doctors and how that affects us as well…that feeling as a GP if you were to find out that the patient that you’d seen was a doctor, it almost gives you a sense of uneasiness. It's almost like you’ve been on a test because they know exactly what and when you should be doing and if you don’t meet their expectations, you can feel a bit inadequate perhaps (ID18)* *I don’t necessarily think that they’re any different or the expectations of me are any different or that even they’re going to get any different treatment. It’s mostly my insecurities around, do I think I’m good enough as a doctor – that tend to creep up to the surface more when I know I’m seeing colleagues and colleague’s families (ID07)* *I think it’s just that interpersonal fear that we all have of a colleague thinking that you’re not very good or that your clinical skills or your diagnostic skills are not that great and the embarrassment I guess of that (ID03)* ***With GP-patients specifically*** *I guess I’m more probably self-conscious about what I do, feeling like you’re perhaps being measured up a little bit to what they do. I feel a little bit more exposed I guess (ID08)* *The bad thing is sometimes I can be a bit more, feel more intimidated or a little bit more not threatened but imposter syndrome. I’m a GP treating a GP like what’s going on here* (ID15) *The ones I find most difficult are the GPs (laughs). And the ones that I find easiest to work with are the specialists. They tend to come in and they’ll often say look I know about this and nothing about anything else. So – and then they’ll sort of revert to the role of a patient as I know it. [….] But I often find GP’s very difficult and I get uncomfortable – you sort of feel they’re questioning everything you do. So, I lose confidence in myself* (ID09) *GPs are more intimidating than a specialist because you think you’ve got this. You know everything, and I do see GPs from country towns who come to see me from an hour or two away, and they are really great GPs, but some of them will argue…*(ID11) *No because I can really put myself in their shoes. I mean I’m in that situation myself not infrequently, and I know how it goes, so and also because I feel pretty confident about my own skills and my own limits of knowledge and scope of practice* (ID17)
*Fear of making an error*	***With doctor-patients generally*** *Another negative might be that there’s perhaps a greater fear perhaps that you might make a mistake or do something wrong. There’s a bit of a cliché that things tend to go wrong a bit with medicos when you’re dealing with – So, there’s perhaps a little bit of apprehension there. And if I do miss something, the consequences can feel greater than with a non-doctor patient, as you have a little less room to hide* (ID21) *There’s a little bit of a pressure if the outcome is not good. I feel like, it’s always going to be a challenging situation but there might be a ramification, I’m not sure if it’s more likely that litigation comes up or more likely that, well, medicine is a small circle. I’m not sure if it’s rational but that definitely crosses my mind* (ID22) *I don’t think it’s worse* (to make a clinical error with a doctor-patient)*, but I think I would probably feel worse and more nervous about the potential outcomes with it and I think it is likely to be on my mind a bit more. I guess it’s that like oh my God is it going to get out, or are they going to tell their colleagues and all those sorts of things. It’s not just, it’s something happening within your circle rather than sort of a separate circle to you. So there’s the consequences to you of making a mistake in a regular patient but then times by the fact it’s someone within your circle* (ID03) ***With GP-patients specifically*** *I think the concerns about being judged for not being good enough would be higher with a GP because they, because they’re more likely to know my friends, and I’d worry a bit more about the reputation of it, or if I’ve done it wrong, I’d worry more about it with GP’s than I would with specialists* (ID16)
*Leading to over-investigating*	***With doctor-patients generally*** *ID05: This might be a fear that we’re under-treating them, so I will over-treat them, I guess, over-investigate…* *CH: So you don’t miss anything?* *ID05: Yes that’s right, and maybe there’s a slight fear of being judged incompetent, or negligent, so we tend to do more tests* *Well there are tests you know that on a normal patient I don’t – I'm confident, I say it is viral infection, I will treat you like that. So it’s a kind of a – I don’t know, it’s over-servicing, adding some tests.[….] To prove that yes, I'm right (ID25)*

The term ‘imposter syndrome’ was used by several participants, and not just junior doctors. These concerns (being good enough, and being seen as good enough) seemed to relate to the fear of losing the respect of a colleague, even of the wider profession.

Concerns to avoid clinical errors, and appearing competent, could lead to over-investigating their doctor-patient.

*There may be some instances where you over-investigate, because you don’t want to be guilty of missing something* (ID02)

Many participants said they felt especially self-conscious, even anxious, about seeing patients who were GPs like them, compared with other specialities. Reasons for this increased discomfort included feeling their care was being assessed, that their GP-patient was comparing the treating doctor’s approach to how they would respond. As members of the same craft group, a clinical error could have a greater impact on reputation amongst close colleagues. Several participants also commented that GPs could be challenging patients, more likely to question the treating doctor’s approach as compared to doctor-patients from other specialities, who responded more like a ‘normal’ patient.

Several participants asked at the end of interviews if their experience of anxiety when treating doctor-patients was unusual and were relieved when told it was not.

*We don’t really talk about this amongst ourselves and it’s actually quite nice to… It’s actually quite reassuring in a way that my anxiety and my fears are shared by a lot of my peers, that’s quite reassuring* (ID22)

### Boundaries and control: how doctors honoured autonomy and navigated role ambiguity

Some participants recognised the challenges of balancing respect/collegiality within the consultation: the need to respond as a doctor and “*honour that autonomy, and also honour the fact that they’ve come to you rather than do it all themselves*” (ID04).

Navigating shared decision-making with a medical colleague could be fraught, where there is a danger of “*leaving it too much up to them, in the guise of believing you’re being collegial, but actually not letting them be a patient*” (ID01). Participants also talked to the challenge they felt when they felt that their opinion was not listened to or followed, describing how this disrespect could undermine the doctor patient relationship.

*If your opinion is not respected or acted upon I do find that pretty challenging (pause). I find it quite hard to have a good, it’s not a normal doctor patient relationship and if you feel unrespected, that kind of undermines the relationship in a profound way* (ID17)

This was evidenced by descriptions of doctor-patients seeking other tests, ignoring advice, not following up or bypassing the treating doctor entirely and self-referring to a specialist.

*My main issue is that most of the doctors I see continue to self-prescribe/self-treat, order their own investigations, arrange their own referrals to specialists etc. Not just for themselves but for their family. It is a real problem in this space, the elephant in the room. It is the number one reason that really makes me hesitant to treat doctors. It is disrespectful to me, and potentially a medico legal issue for me too, as I am hamstrung in my management due to being cut out, and dangerous for them and their family* (ID11)

While the term ‘respect’ was common, these comments also appear to reflect a fear of losing control, or a fear of being ignored, threatening the treating doctor’s sense of professional competency. As the treating GP, your usual patient can decide to seek another opinion from another GP and get the referral they want, but your doctor-patient can actually just go and do what they like with the treatment/investigation/referral – even if they shouldn’t – and it feels quite upsetting to the treating GP when that does happen.

Doctors can find accepting the role as patient and relinquishing their role as doctor challenging. Participants were conscious that doctor-patients might have treated themselves, and they may have already determined their own diagnosis and decided on a plan for management. The doctor-patient’s struggle with their role reversal presented challenges to the treating doctor. Participants described needing to navigate this space, sensitively putting their diagnostic perspective and clinical judgement into the mix when providing care.

*Interviewer: So it’s something about the – you used the word ‘autonomy’, ‘control’ is a bit stronger, but like ‘who’s in control here?’*

*ID04: Yeah, yes yes yes (emphatically)- and I know that medicine now is all about partnerships and being patient-centred, […] but certainly that, that dynamic is different for sure.*

Table 4 reflects three different approaches participants used to navigate the role ambiguity within the consultation and set boundaries.

**Table 4:** Ways of establishing boundaries in the consultation

Strategies used	Exemplar quotations
Treat them like a colleague	*OK you’re a doctor, so I’m just going to, you know I’m going to use a few medical terms and kind of go through things a little bit quicker, but just pull me up if you think I’m talking about something you don’t know or if you don’t feel comfortable* (ID20)
Check if they want to be treated like a usual patient	*I’ll have a spiel about “I know that we are in the same field, I’m open to whatever arrangement that you prefer. If you would like to be treated like any other patient, very happy to do that. If you feel like I’m over explaining things…”[….] Almost every single time, actually every single time the response is “Oh right, thank you for asking, just like everybody else”* (ID22)
GP sets clear boundaries	*Often I have to establish that boundary the very first time I meet them. I say look how do you want to play this? I’m the GP, I’ll probably do it my way and if you disagree come in but I’ll probably, I’m going to be a GP and you can be the patient, let’s try, see how it goes* (ID15)

### Impact of years of experience on emerging themes

While there was a wide of range of clinical experience amongst participants, there was marked consistency in responses relating to all three main themes: challenges with navigating boundaries, the need to demonstrate respect, and the apprehension felt by many when treating a doctor-patient. Several of the most experienced GPs still spoke of feeling anxious about being judged on their competence, and acknowledged they might order more tests to ensure nothing was missed. They also acknowledged they might avoid sensitive topics, like substance use and mental health during history-taking, though they were more likely than the younger participants to attribute this avoidance to their previously-only-subconscious assumption that ‘they are like me’ and therefore would not be experiencing such issues.

### Doctor-patients not disclosing they are a doctor

GPs were asked if they were aware that some doctor-patients might not disclose they were a doctor, and how they felt about that. Most found it easy to imagine why a doctor-patient might not disclose this: “*They don’t want to be treated differently*” (ID06), but this could complicate the consultation.

*I think it’s difficult if you conduct the consultation all the way through and at the end they say ‘actually I’m a haematologist’ and you think ‘you probably should have told me, cos that probably would have changed some of the communication’, but at the same time we need to be conscious that we shouldn’t treat people any differently, and still explain things in plain language and be clear in how we step through the consultation* (ID04)

Similarly, the treating doctor could feel that their collegiality was challenged if their doctor-patient doesn’t tell them they are a doctor.

*I think it’s important to identify yourself as one out of respect for the clinician. Because I feel as for me being the clinician I like to know that they’re a doctor…. I would be embarrassed if I only found out at the end of a consultation that someone was a doctor* (ID10)

Yet nearly a quarter of participants admitted that they themselves (as patients) had, on occasion, not disclosed that they were a doctor.

### Mental health consultations

Mental health issues were identified as a particular challenge for GPs when treating doctor-patients. They were aware of the fears a doctor-patient might have in disclosing mental health concerns. Participants talked about behaving more ‘collegially’ than ‘professionally’ on these occasions. Sometimes they did not ask specific questions related to depression/suicide for fear of offending their colleague or out of concern they may need to report to the Medical Board. Two participants indicated they would tell their patient to stop discussing an issue if it sounded like they may say something that needed reporting. Some GPs were concerned that they could become the sole mental health care provider if a doctor-patient refused to see a psychiatrist, likely due to pervasive mental health stigma [25]. Others recognised that help-seeking could be hard and wanted their doctor-patient to feel safe in seeking help and felt that supporting them through mental health issues was rewarding.

*Sometimes it’s stigmatised, if they have a mental health condition, or if they’re sick- you know doctors are not supposed to be sick, are we? I want to get rid of that stigma, and I want to help them feel comfortable [….] And heard in the conversation* (ID05)

### Billing doctor-patients

Most participants followed a long-held, mostly unspoken, tradition of extending professional courtesy to medical colleagues by only charging the amount that the treating doctor would receive from Medicare (the publicly funded universal healthcare insurance scheme in Australia). This meant that the doctor-patient paid nothing out-of-pocket for the consultation. However, many who extended this courtesy also questioned this approach. There were financial implications for the treating GP:

*Well I do feel the onus to bulk bill all my colleagues, but you know some of them earn much more than I do. [….] I want to bulk bill my colleagues. And that wouldn’t be a financially good thing to me in the long run obviously. Especially because they don’t need to be bulk billed. Whereas I’ve got patients who are homeless. Who can’t afford a meal* (ID23)

*It’s tradition. The trouble is in general practice it doesn’t really stack up because it’s, because their rebates are so discrepant and these guys, they’re hard work, seeing doctors* (ID08)

Some said this de-valued the medical service and could reduce the respect for the care. Others believed it can lead to fewer or shorter appointments.

*To be honest I think it would be better to be private billing because I think it means that people are valuing the care that they are getting, and not just assuming I’m going to not have to pay for this* (ID06)

*I think there are really good arguments for paying as the doctor-patient. Like you want to get treated the same. You don’t want to financially inconvenience the person who is seeing you. You don’t want to feel like you are burdening them, and therefore you know I think actually if you didn’t pay it might put you off seeking medical attention if you need it* (ID17)

Most participants indicated that billing was never discussed with their doctor-patient and they did not know how other GPs in their practice billed their doctor-patients.

### Positives of treating a doctor-patient

There were positive aspects of treating doctor-patients. There were perceived benefits in working with a patient with a high level of health literacy and shared language. It was rewarding and meaningful to be *“providing care to people who we know have trouble accessing healthcare systems”* (ID04), and *“who tend not to look after themselves”* (ID20).

*When I see junior doctors having a hard time in the hospital system, I feel quite like a good advocate for them […], reality checking some of the things that they’re negotiating and why they’re not necessarily thriving and broadening that out to being about the system rather than necessarily about their own personal resilience, I find that quite I don’t know, quite (pause) meaningful to have those conversations* (ID08)

### A grounded theory model of how GPs experience and respond to patients who are also doctors (see Fig. 1)

**Fig. 1:**
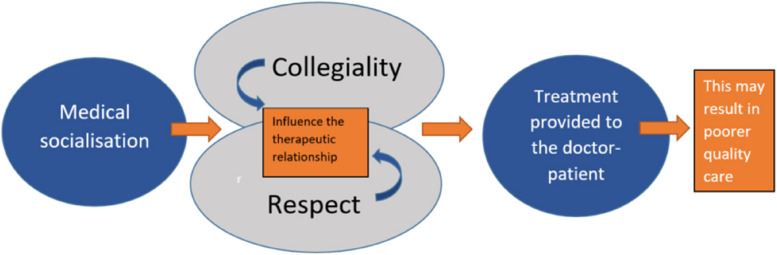
A grounded theory model of GPs’ experiences of treating doctor-patients

Despite elaborating a number of ways that they had made exceptions to the rule of treating doctor-patients like any other patient, participants still stated that this would be their advice for a new GP registrar about to see their first doctor-patient. This perpetuates the myth that this is a normal consultation. Using grounded theory methodology, we have developed a model in which the professional socialisation of doctors is central, highlighting the importance of maintaining collegiality and demonstrating respect for colleagues. When treating doctor-patients, it is challenging to adhere to these professional values while maintaining appropriate boundaries that enable objective care. The desire to assure respect and collegiality impacts how the treating GP cares for the doctor-patient, so it is not possible to care for them as a normal patient. This could have positive or negative effects on the treatment. When not recognised, this could result in poorer quality care.

## Discussion

This investigation into the experience of doctors treating doctor-patients provides a deeper understanding of the dynamics of this potentially challenging therapeutic relationship when GPs are asked to be a doctor’s doctor. Participants described how the care they provided to doctor-patients was different to other patients.

These findings have informed a model (see Fig. 1) designed to help GPs visualise how medical socialisation can impact the care that the treating doctor provides to doctor-patients. Collegiality and respect sit at the centre of the experience of treating a doctor-patient and shape the consultation.

Collegiality changed the process of engagement through their shared language, assumptions about expectations of care and reluctance to explore sensitive issues. When delivering ‘normal care’, the treating GP is constantly navigating collegiality through language, through shared expectations, through recognition of shared experiences and understandings of a shared landscape (including medicolegal landscape). Respect resulted in concerns about offending the doctor-patient with sensitive questioning, pressure to avoid clinical errors and challenges in boundary setting.

The findings of this study highlight how professional socialisation can influence the doctor-patient’s care without being explicitly acknowledged during the consultation. There was an instinctive desire to meet normative expectations. To ignore these complex dynamics of the doctor-doctor consultation is to ignore the Elephant in the Room. This metaphor, used by several of the participants, fits well.

Medical culture molds trainees’ values and behavior through informal and tacit modes of professional socialisation. The socialisation process is partly learned through the hidden curriculum [26] which refers to what is implicitly taught as doctors-in-training observe senior colleagues at work [27]. The sense of collegiality is likely to be stronger with those in the same speciality which may explain the greater anxiety when treating another GP. The doctor-patient has the capacity to challenge the treating doctor’s authority in the therapeutic relationship in unique ways. The treating doctor relies on the doctor-patient to participate in the consultation by adopting the patient role. Junior doctors and specialist doctors may accept this patient role more readily. GP-patients were considered more likely to challenge this role.

If you are strongly socialised into a powerful role, with the expectations of being highly trained and knowledgeable, then the role reversal can be challenging. While this study focused primarily on the experiences of the treating doctor, many participants also spoke about their experiences as a patient. It seemed these two sides of the same coin could not be compartmentalised when talking about a doctor-to-doctor consultation. Kay et al.’s [7] qualitative study exploring what doctors want within a consultation found that participants often “illustrated their ideas about how a treating doctor should consult by giving examples of their experience as a patient”. However, while they found a mirroring of expectations between the treating doctor’s perspective and the doctor-patient’s perspective, this was not always the case here. GPs sometimes articulated an unhappiness with what they have experienced as a patient and acknowledging how, as the treating doctor, they might struggle to provide the kind of care they would like to receive.

This study highlights that being a treating GP is challenging. Yet the ‘rules and guidelines’ available for treating doctors fail to acknowledge these aspects of the consultation. These findings challenge the current guidelines and demonstrate the need to overtly recognise why the doctor-doctor therapeutic engagement is different. In doing so, it will be possible to effectively address the Elephant in the Room through better education of the doctor’s doctor.

### Strengths and limitations

This study offers a rich understanding of the experiences of the treating GP caring for doctor-patients. These findings are consistent with previous research and extend the current understandings by providing robust insights that enabled the construction of a model to inform care for doctor-patients.

A number of limitations are acknowledged. First, the GPs who chose to participate may have had strong views about caring for doctor-patients, potentially driven by past negative experiences. While this is possible, at least two participants stated they had chosen to participate because they wanted to promote both the rewards and the importance of being a “doctor’s doctor”. Most participants were metropolitan GPs with fewer regional GPs being recruited (see Table 1). Regional doctors in Australia work in small communities and face additional challenges of isolation that can impact access and confidentiality. Only GPs were asked to participate, but other specialists care for doctor-patients and there would be benefits to broader the recruitment in future studies.

While the interviewer’s position of ‘not a doctor’ may have created a more neutral environment, avoiding unspoken assumptions, it is possible that not being a doctor increased the chance that some interviewees were cautious about what they disclosed, to avoid misunderstanding by a non-peer and sharing only ‘safe’ information.

### Comparison with existing literature

Hutton et al.’s (2023) scoping review found few empirical studies that have explored the treating doctors’ perspective of physician health [18, 28–31]. Role ambiguity, assuming the patient has sufficient knowledge, and anxiety about scrutiny of treating doctors’ performance have all been noted previously. Only two of the empirical studies [18, 28] asked doctors what they do differently when treating doctor-patients. While physicians in Avinger et al.’s [28] study within a specialist cancer centre said they would offer the same tests/treatment to doctor-patients as they would to other patients, the findings in Teng et al.’s [18] study (with primary care physicians) were comparable to ours, in that some doctors acknowledged they would both order more tests/procedures, and communicate results differently with their doctor-patients. While Teng et al. found that experienced physicians were less likely to report anxiety, this was more variable for our seasoned GPs, and the wish to show respect to colleague-patients was for some a powerful if at times subliminal force.

While other studies have used a broader socio-cultural lens and acknowledged the impact of medical culture and professional identity [7, 32–37], little has been previously written on how medical socialisation and culture impacts the care delivered to the doctor-patient.

Domeyer-Klenske and Rosenbaum [29] found that doctors typically adopt one of three strategies: ignoring the fact that the patient is a doctor, allowing the patient to take control of the treatment, or acknowledging that the patient is a doctor and negotiating their medical care. The interviews in this study revealed a similar pattern. The third strategy is an approach that acknowledges the Elephant in the Room.

### Implications for practice

This study, a collation of voices of the treating doctor, addresses an important gap in the literature highlighting the impact of professional socialisation on the complex dynamics of the therapeutic relationship. By naming the Elephant in the Room, this study challenges the current guidelines for doctors treating doctor-patients with their rather simplistic ‘keep it normal’ recommendations that fail to acknowledge the reality of how these dynamics impact the delivery of care to doctor-patients.

We argue that better guidance is needed to enable treating doctors to navigate this important role of caring for their doctor-patients. Beyond the distillation of its findings, this study presents a pragmatic model that can support future training. The model highlights the need to overtly acknowledge the complexity of the doctor-doctor consultation. When training doctors for their role as treating doctor, it is vital to enable them to effectively manage the issues of collegiality and respect. Such education is relevant at all levels of medical training. Even medical students need to recognise that they will be treating doctors in the future. Although being a doctors’ doctor is relevant to all specialities, all doctors should have their own general practitioner. Therefore this study is especially relevant to general practice training. Improving the care provided to doctor-patients has important implications for physician well-being.

## Supplementary Information

Supplementary Material 1.

Supplementary Material 2.

## Data Availability

Data is provided within the manuscript or supplementary information. To protect study participants' privacy, raw data is not able to be shared openly.
