# Supplementary material for: “Do they think I’m good enough?”: General practitioners’ experiences when treating doctor-patients
Source: BMC Prim Care. 2024 Sep 16;25:340. doi: 10.1186/s12875-024-02592-1 (PMC11406865; doi:10.1186/s12875-024-02592-1)
Supplement: Supplementary file 1 — Supplementary Material 1. [file 12875_2024_2592_MOESM1_ESM.docx]

Supplementary material 1

**GP experiences and challenges in treating doctor-patients**

Semi-structured interview guide

Questions in red font added for later interviews, based on the subject area being raised by early participants and seen by interviewer as key to the emerging theory

**Topic/Thematic area 1 – Context of practice and any special interests**

Prompt – How long have you worked as a GP? In what kinds of practice?

Prompt – Do you have areas of speciality?

**Topic/Thematic area 2 – GPs feelings and experiences in treating other doctors**

Introductory prompt – You may have heard the saying “Doctors make bad patients”. What do you think about this?

Some doctors report that when they need to seek medical help, they don’t disclose that they too are a doctor. What do you think about this?

Prompt - What is hard/challenging about treating doctor-patients?

What do you like about it?

Has this changed over time?

Does it feel any different for you, if your doctor-patient is a fellow GP, or from a different speciality?

Prompt – Role reversal

Do you try to address this dynamic directly? If so, how?

Would you generally find a way to acknowledge the possible discomfort of being a doctor-patient (or only if your doctor-patient raises it?)

**Topic/Thematic area 3 – Clinical practice issues treating other doctors**

Prompt - Do you treat your doctor-patients the same as other patients? Is this what you aim for? In what ways might you treat them as colleagues?

Prompt - Are there issues you hesitate to ask about?

Prompt -Do you routinely ask about mental health? AOD use?

Prompt - Does the possibility of AHPRA notification issues mean you might hesitate to ask certain questions?

Prompt - Do you ask if your doctor-patient self-treats/ self-prescribes?

If not mentioned by interviewee:

Do you treat payment differently for a colleague?

If so, why?

Do you think confidentiality is a bigger issue when your patient is another doctor?

**Topic/Thematic area 4: Reflections on being a patient.**

Prompt - Do you have your own (independent) GP?

- - If not, why not?
  - If yes, how did you choose them?
    - What are your expectations from your GP (e.g. professionalism, empathy etc)

Prompt - If you have sought medical treatment from other GPs, do you consciously try to replicate what was helpful for you, with your own patients?

**Topic/Thematic area 5:**

**What advice would you give another GP seeing a doctor-patient for the first time?**

**Is there anything that is important that we haven’t talked about?**
