# Supplementary material for: “Do they think I’m good enough?”: General practitioners’ experiences when treating doctor-patients
Source: BMC Prim Care. 2024 Sep 16;25:340. doi: 10.1186/s12875-024-02592-1 (PMC11406865; doi:10.1186/s12875-024-02592-1)
Supplement: Supplementary file 2 — Supplementary Material 2. [file 12875_2024_2592_MOESM2_ESM.docx]

Supplementary material 2

Coding categories

Open code axial code selective code

| Am I good enough? Imposter syndrome  Wanting to be thorough  Wanting to be seen as knowledgeable, competent/ concern about being judged | Need colleagues to see me as competent | Apprehension |
| --- | --- | --- |
| Fear of mistakes  Embarrassment | Not meeting DP’s expectations | Losing respect |
| DP ordering own tests  Not following up  Self-treatment  Self-diagnosis | Harder to manage/navigate overall care | Respect  Power/control |
| Bypassing GP, going directly to specialist  Only seeing GP for referral to specialist | Not being shown respect as treating doctor, or respect for speciality | respect |
| Bulk billing | I need to show respect to my DP | respect |
| Use of medical language |  |  |
| Self-diagnosis prior |  |  |
| Important to acknowledge they are also a doctor |  |  |
| DP wanting to self-manage | Might not align with what I think should happen | Power  Control |
| Impact of age/experience | Does DP feel they need to be in control?  Ability of DP to change hats / Role reversal |  |
| Use of medical language | A way to demonstrate collegiality | Collegiality |
| Important to acknowledge they are also a doctor |  |  |
| Avoid Mental health questions  Avoid Substance abuse questions | Don’t want to offend  Don’t want to jeopardize relationship  Don’t think they’re relevant | Collegiality |
| Over-testing | Fear of making a mistake  Wanting to be seen as competent | Loss of respect  Competence |
| Mental health AHPRA- hesitation to ask- questions of competence | Don’t want to jeopardize relationship- | Collegiality |
